# A Few Considerations on the Leptothrix Buccalis

**Published:** 1897-12

**Authors:** T. Choquet

**Affiliations:** Paris


### Abstracts and Translations.

### A FEW CONSIDERATIONS ON THE LEPTOTHRIX BUCCALIS.

BY T. CHOQUET, D.E.D.P., PARIS.

In a paper presented in my name and in that of Mr. Grimbert at the Dental Congress held at Bordeaux in 1895, I mentioned, among the microbes I had succeeded in isolating from the mouth at the same time as the coli bacillus, the presence of a leptothrix buccalis. The separation and the obtaining in pure culture of this microbe was effected by the use of the phenicated bouillon, which Dr. Peré had suggested for the study of the coli bacillus. Since then I have continued my researches, and I have to-day the honor to submit to you the few observations I have been able to make.

By the method of the phenicated bouillons one can isolate in pure culture from the buccal cavity several species of leptothrix, differing from each other either by their biological and morphological nature, or by their chemical reactions. But, first of all, what has until now been and is still understood by the denomination of leptothrix?

Among the numerous species which Leeuwenhoek was the first to discover in the saliva (though at that period the microscopic investigation was no easy matter), he had signalled the presence of a segmented thread of great length and without movement, or rather, presenting an almost imperceptible oscillating motion. Robin had studied this microbe and had at last given a good description of it from a purely microscopic point of view. It is this author who first attributed to it the genesis, the formation of the other microbes which are met with in the mouth.

This theory has been taken up some years since by a learned Italian, Filandro Vicentini. While rendering due honor to the care taken by him in his experiments and researches we cannot partake in his ideas. There may be some truth in the theories which he maintains, but bacteriology is a science of too recent date, and until the contrary be proved we shall consider the leptothrix as a peculiar species, a pure species.

According to the definition given by Professor Flügge, the species which interest us belongs to the leptothricæted class, and show filaments with or without sheath, the division of which does not extend far, and of which the cells do not contain any sulphur. These filaments are sometimes isolated, at others grouped in bunches, in tufts, entangled in each other, as one can see in examining certain advanced carious cavities, the walls of which are sometimes lined with these tufts.

The direction of these filaments is not always straight, it is even slightly waving. According, too, as one has to do with one or another kind cultivated in a nutritive medium, or in a different one, one will find one's self in contact with thin and lengthy filaments, or with short and thick ones.

The leptothrix are easily stained by most staining agents. They take the Gram method, which imparts to this microbe a fine black-violet tint. On attentive observation of certain species, spores in course of formation may be distinguished.

The following is the receipt for a staining solution which I have made, and which, when followed by impregnation, according to Van Ermengen's method, shows very distinctly the spores of the leptothrix buccalis, and of most microbes growing by sporulation:

| | | |
|---|---|---|
| Victoria blue.................................... | gr. | 0.25 |
| Methylene blue.................................. | " | 0.25 |
| Absolute alcohol................................ | " | 25.00 |
| Distilled water................................... | " | 125.00 |

When the preparation is fixed on the glass slide a few drops of the above solution are poured on, and one can, if one wishes, heat it for five or six seconds over a small Bunsen-burner flame until the vapor subsides. Wash well and continue with the silver method, and a result can be obtained in two or three minutes.

Does this mixture act as a corrosive? I do not know; but, thanks to it, Van Ermengen's method is greatly simplified, and the spores are distinctly apparent in black in the interior of the light or dark-blue tinted filaments.

A thorough reaction, which Miller was the first to give, is the reaction of iodine, combined with some acid, even a weak acid, which reaction causes the microbe to assume a violet tint, and allows it to be distinguished from most of the other microbes which are met with in the mouth and which do not present this reaction.

We know that the leptothrix is found in the buccal cavity; but which is its favorite abode, and can it be met with elsewhere? Its favorite abodes are the dorsal surface of the tongue, the dental interstices, and the tonsils; also sometimes in carious teeth, where it is met with in tufts in the margin or in the bottom of the cavity. It is also found, but by chance only, in the concretions of the lachrymal pipes (Jaffé), in the expectoration of pulmonary gangrene (Leyden, Craube). Weigert has also met with it once in an abscess of the tongue.

It is normally met with in the mouth of healthy persons, but it is very difficult of cultivation on account of the sort of microbic antagonism which appears to exist between this species and the other species which compose the microbic flora of the mouth. These species stop and choke the development of the leptothrix. It is to be met with—and this is an entirely personal opinion—very frequently and in an almost pure culture in the mouth of idiots, of weak-minded persons. In this case *the saliva* is literally filled with it. In the mouth of three idiots of eighteen, twenty, and twenty-seven years of age residing at home, and of whom no care was taken, including no special care of the mouth, I have found it *three times*, and not in the tartar nor in the amygdalin crypts, but simply in the saliva reposing on the point of the tongue. It is with these three cases that this paper is concerned. I wished to undertake further researches in lunatic asylums, and thanks to the kindness of Dr. Bourneville we have been able, my colleague Dr. Durand and myself, to make at Bicêtre in the lunatic section about one hundred experiments in connection with the study of the leptothrix. We have, unfortunately, met with an almost total failure, on account of the too great cleanliness of the patients' mouths. Every morning the inspectors force the children to clean their teeth, and rare are those in whose mouths tartar can be found.

To run a chance of finding leptothrix in an idiot's mouth, or in one of feeble intellect, it is necessary that the health of the mouth be less cared for. Does the general state favor the increase of the leptothrix to the exclusion of other microbes? I leave to others the care of developing this question and giving a reply.

Besides the human mouth it is also found in the buccal cavity of most animals, such as dogs, sheep, and oxen. Miller has found it in the mouth of a dog suffering from pyorrhœa alveolaris. To this species he has given the name of leptothrix gigantea. Dr. Phisalix, professor at the Museum of Natural History of Paris, has assured me he had found a species of leptothrix in the throat of a stork. From this it is observable that this microbe may be met with in almost all mouths. When it is met with in the human throat it is on the lateral surfaces of the tonsils in the form of small yellowish spots, easily detachable by the platinum wire used for the inoculation.

Has the leptothrix a pathogenic action?

It would appear so, according to Leber, who, by inoculating it on the cornea, is said to have produced on the latter a serous suppuration. But Leber does not say in his remarks whether he inoculated a pure culture of leptothrix or if he inoculated only a small quantity of buccal mucus. It would rather appear that this latter assertion is the true one, for most authors agree in saying that the leptothrix is not cultivable. Vignal and Rasmussen alone, eight or ten years ago, succeeded in studying it in pure culture. Rasmussen appears to have attained this by means of gelatin and potatoes. Unfortunately he gives no details of the method which he used.

Vignal (*Archives de Physiologie*, 1887), after numerous experiences made in 1885–87, has given an excellent definition concerning the species, and also of the morphological characters. He was, we believe, the first to draw the attention of bacteriologists to the difficulty of cultivation of the leptothrix, and especially of its isolation from the other species.

For my part, I am absolutely persuaded that he had studied one species only, for the result which he gives is different from that obtained by me. Moreover, it proves most surely that there is not only one kind of leptothrix, but different sorts, as you will soon be able to judge.

He advised the inoculation to be made with dental mucus and tartar very much diluted. The following are the observations he had made in the different mediums of cultivation: (1) *Bouillon.*—The leptothrix disturbs it slightly and forms a white deposit at the bottom of the tube. (2) *Gelatin in puncture.*—Boil culture with a head and paint. Liquefaction like a funnel at the end of three or four days. (3) *Gelatin in plates.*—The colonies make their appearance at the end of three or four days in the form of whitish spots, denticulating about the fifth or sixth day. They then become of a dirty white with an opaque centre and semi-transparent border. Then the gelatin becomes soft and ends by becoming perfectly liquid. (4) *Potatoes.*—Dirty white spot growing very rapidly. (5) *Agar-agar.*—35°–38° C. The inoculations appear at the end of twenty-four hours. Same aspect as on gelatin.

Vignal added that on microscopic examination he had been able to see very distinctly with a high magnification in the inner part of the filamenta the presence of transversal sections on the preparations stained by aniline colors. In this, he differs totally from Robin, who said that *no* trace of articulation was *ever* met with in the filaments.

Here, then, is another proof that there is more than one variety of the microbe which interests us. And this is absolutely the opinion of Miller, who, as you know, has carefully studied the question, and is in a position to speak learnedly on the matter.

Here is the description of the three cases in which I have succeeded in obtaining in *pure culture* the leptothrix.

I specially draw your attention to the difference existing between these species.

CASE I.—Miss H., aged eighteen. Insane. Very bad state of mouth. Soft tartar. Hypertrophied gums. Mucus taken on the tip of the tongue. (1) Inoculation in phenicated bouillon, which is put in the incubator at 36°–37° C. *Four hours* later complete disturbance. Second inoculation with this bouillon in another tube of phenicated bouillon. Four hours later, complete disturbance. A plate of gelatin in Petri's box is made with this second result. At the end of two days whitish colonies, causing *no liquefaction* of the gelatin, these colonies spreading more and more without liquefaction. (2) *Ordinary bouillon.*—Complete disturbance at the end of four or six hours. The bouillon remains dense, and shows at the end of two or three days a *thick film.* No clearing. (3) *Lactose.*—Steady fermentation. *Peptone.*—Nothing. *Potatoes.*—Thick, whitish trace. *Microscopic examination.*—Long segmented filaments, presenting very *clearly defined articulations.* Takes the Gram and all aniline dyes. Examined without staining, in damp chamber they present a to-and-fro movement barely visible. Purple coloration by iodine and acetic acid.

CASE II.—Mr. Z., aged twenty. Idiot. Appears but twelve years old. Mouth in a very bad state. Saliva taken for inoculation. (1) *Inoculation in phenicated bouillon.*—Dense after twelve hours. Presents the same character as that described by Vignal, as also the liquefaction which No. 1 did not produce. (2) *Lactose.*—Nothing. (3) *Peptone.*—Nothing. (4) *Potatoes.*—Whitish trace.

CASE III.—Miss P., aged twenty-seven. Idiot. Very bad state of the mouth. (1) *Inoculation of saliva in phenicated bouillon.* Incubator at 36°–37° C. Disturbance after six hours. Thick film on the surface. (2) *Gelatin in plate.*—Same colonies as in No. 1

without liquefaction, even at the end of three months. The colonies appear from twenty-four to forty-eight hours. (3) *Lactose.* —Very defined fermentation. (4) *Peptone.*—Reaction of indol with the azotite of potash and sulphuric acid. (5) *Potatoes.*—Hardly visible traces. *Microscopic examination.*—Voluminous filaments presenting a well-defined segmentative and spores brought to light by the blue solution which I have mentioned, followed by impregnation of nitrate of silver. The envelope does not take color. Here, then, there are three different species of leptothrix, resembling each other in the appearance of their colonies, but differing from each other, either by the form of the microbe, or, and above all, by the nature of the cultures. In one case the gelatin is liquefied, in the two others it is not.

In two cases we find fermentation, whereas in the other we have none. Lastly, one case gives us the reaction of indol, while the two others do not. I think it is not necessary to add more to show the plurality of the species of leptothrix. This work is certainly not complete, I allow, since I have not been able to ascertain the degree of pathogeny which the species possess; but I wish to present to you these results, and hope at a future Dental Congress to acquaint you with any more results that I may obtain.

To conclude, I wish to draw your attention to the similitude of the colonies that three different sorts of microbes can present which might be confounded with the leptothrix. These are the zopfii, the coli bacillus, and typhic bacillus, but especially the zopfii. It is true that a simple examination with the microscope would suffice in such a case to avoid confusion with the leptothrix, being all three simple bacilli, and not attaining the tenth part of the length of a filament of leptothrix.—*Journal British Dental Association.*

